# Surgery

**Published:** 1824-03-01

**Authors:** 


### Surgery.

1. *Surgical Operations.*† In the 13th number of this Journal we stated the objects and use of the little volume whose title page is annexed, and promised to give some extracts from it in a succeeding number. We now proceed to fulfil our promise. It is hardly necessary to premise that the few extracts we make will be entirely confined to such modes of operating as our author witnessed or learnt on the Continent:—and first of 1. Œsophagotomy. The following is the plan recommended by Lisfranc, in cases of foreign bodies being lodged in the gullet, and incapable of being withdrawn by fingers or forceps.

"The patient should be seated in a chair, with his head reclining backwards on the breast of an assistant; the operator placing himself in front, takes the scalpel or bistoury, and, holding it like a pen, commences his incision on the inner border of the left sterno mastoid

† A short Treatise on Operative Surgery, describing the principal Operations as they are practised in England and France. Designed for the Use of Students in operating on the Dead Body. By Charles Averill, Surgeon, Cheltenham, &c. London, Jackson, 1823. Duodecimo, pp. 172.

muscle, opposite the superior edge of the thyroid cartilage, and continues it down to the lower edge of the cricoid. An assistant now draws the carotid sheath to the outer edge of the wound to secure it from the knife; while the operator, cutting carefully through the cellular tissue, exposes the œsophagus, where it inclines to the left side from behind the trachea. A canula with a grooved stilet, or the sonde à dard formed like a female catheter, but considerably longer, is to be passed by the mouth down the œsophagus, inclining its point to the left side, which causes it to be readily felt from the external wound. The stilet is now to be pushed forwards through the coats of the œsophagus, when the operator feels with his finger along its concave edge, to ascertain that no large arterial branch be situated on it, and then passes a bistoury into the groove, which directing it onwards opens the œsophagus. He now feels for the foreign substance, which is to be extracted by a pair of dressing forceps passed along his finger." 50.

2. *Wry Neck.* A little girl, 10 years of age, was admitted into the Hotel Dieu under M. Dupuytren, early in January 1822, whose neck, or rather head, had been awry for three years, owing to a permanent spasmodic contraction of the sterno-cleido-mastoid muscle of the right side. On the 16th of the same month M. Dupuytren operated in the following manner:—

"The patient reclining against an assistant, a puncture was made, with a straight narrow bladed bistoury, through the integuments just on the inner border of the sternal extremity of the contracted muscle. The blade of the bistoury, being flatly opposed to the muscle, was pushed cautiously behind it, the point being directed forwards and outwards till it protruded just on the outer side of the clavicular border. The edge of the bistoury was then turned towards the muscle, and a sufficient quantity of its posterior fibres cut to allow of the head being placed erect: the instrument was then withdrawn.

"In this way the integuments escaped being divided, and a future scar was prevented; a very desirable object, the patient being a female.

"The cut edges of the muscle were kept asunder by depressing the clavicle, and inclining the head to the left side. The former was effected by binding the right hand firmly to the foot, the knee being bent; thus the clavicular fibres of the deltoid drew the bone downwards; the latter by a roller passed round the head and under the left axilla.

"The patient was kept in bed; and at the end of thirteen days the punctures were healed, and she had free motion of the neck, though from long continued habit she still turned her face to the left side. The bandages were reapplied, and the same bodily position maintained till the twenty-first of February, when they were finally taken away, and the patient pronounced cured, the head being but very slightly inclined to the right side, and having free motion in every direction." 64.

3. *Amputation at the Shoulder-Joint.* As there are some surgeons whose great delight is in operating against TIME, and who always place a stop-watch on the table when they commence, we shall indulge them with the account of a mode of amputation at the shoulder-joint, by which, Richerand asserts that "a dexterous operator can separate the arm from the trunk as quickly as an expert carver detaches the wing of a partridge." The following are Lisfranc's directions.

"Supposing the left extremity is to be removed; the patient is placed on an elevated seat, one assistant pressing the artery above the clavicle on the first rib, whilst another draws the arm forwards. The operator standing behind the patient, with a long bladed catling, pierces the integuments on the inner edge of the latissimus dorsi muscle, opposite the middle of the axilla, and pushes it obliquely upwards and forwards, till its point strikes against the under surface of the acromion; then by raising the handle of the knife its point is lowered, and protruded just before the clavicle, at the part where it joins the acromion. He then, by cutting downwards and outwards, forms a flap from the superior and posterior part of the arm, including the whole breadth of the deltoid muscle, and a part of the latissimus dorsi. This being held back by the assistant, the joint is cut through by passing the knife between its articulatory surfaces from behind forwards, and a corresponding flap is formed by cutting downwards and outwards between the muscles and bone on the inner side of the arm. The vessels being tied, and the flaps placed in contact with each other, the operation is finished.

"In operating on the right side, the patient should be seated on a low chair, and the catling thrust from above downwards, introducing it just before the point where the clavicle is connected to the acromion, and raising the hand as it is thrust backwards and downwards, till it appears on the inner edge of the latissimus dorsi, when the flap is to be formed, and the operation continued as before." 136.

We recommend this little pocket volume to the student, as a guide in operating on the dead body, preparatory to operating on the living.

---

2. *Amputation at the Hip-Joint.* This bold and desperate operation has been successfully performed in September last by Mr. James Syme, Lecturer on Anatomy in Edinburgh.

The subject was a lad about 19 years of age, who had necrosis of the femur for some years, attended with great enlargement of the thigh, and numerous openings through which matters of different kinds were poured out. The swelling extended to within an inch of the trochanter major—emaciation was making rapid progress—and the lad's destruction seemed inevitable, unless an operation was risked. The steps of the operation we find it so difficult to abridge that we shall give them in the author's own words.

"Having, with some difficulty, placed the patient upon a table, so that the affected limb was perfectly free, and ascertained that Mr. Liston was ready to make pressure when and where required, I introduced a narrow knife, about a foot long in the blade, which was sharp on one edge only, at the proper place for transfixing the limb; but being prevented by the bent position in which, owing to long habit, the patient obstinately retained it from passing onwards in the direction of the tuberosity of the ischium by the neck of the femur, I lost no time in the repetition of fruitless attempts, but instantly changed my plan.

"Without removing the point of the knife, I brought down its edge obliquely, and, by a sawing motion, quickly cut back, in a semicircular direction, to the tuberosity of the ischium, up along the femur, and round the trochanter major, so as to form very speedily identically the same flap which would have resulted from the plan I meant to have followed.

"While Mr. Liston covered the numerous cut arteries with his left hand, and compressed the femoral in the groin by means of his right, I gathered together all the mass of undivided parts on the inner side of the thigh with my left hand, and then insulated the neck of the bone by passing the knife close past its lower surface. I now cut close down along the bone for some way below the trochanter minor, and, lastly, made my way outwards obliquely, so as to form a good internal flap.

"Mr. Liston holding aside the flaps, I made a single cut with my long knife upon the head of the bone, which started with a loud report from its socket as soon as abduction was performed. Finally, I passed the knife round the head of the bone, cut the triangular and remaining portion of capsular ligament; and thus completed the operation, which certainly did not occupy, *at the most*, more than a minute.

"I then proceeded, without delay, to take up the arteries, which were tied by our very promising pupil, Mr. Thomas Evans.

"As soon as the femoral, which had been completely commanded by pressure in the groin, was secured, Mr. Liston relaxed his hands in order that we might form some estimate as to the size and number of bleeding vessels; and then, had it not been for thorough seasoning in scenes of dreadful hæmorrhage, I certainly should have been startled, prepared as I was to expect unusual vascularity, owing to the extensive action so long carried on in the limb.

"It seemed indeed, at first sight, as if the vessels which supplied so many large and crossing jets of arterial blood could never all be closed. It may be imagined that we did not spend much time in admiring this alarming spectacle. A single instant was sufficient to convince us, that the patient's safety required all our expedition; and, in the course of a few minutes, hæmorrhage was effectually restrained by the application of ten or twelve ligatures.

"The flaps were now brought together, and retained in contact by means of five or six stitches. Some dry caddis was laid over the wound; and, lastly, I applied a single-headed roller obliquely round the body and stump, moderately tight, so as to afford proper support to the flaps; and then we lifted into bed the patient, who was wonderfully little exhausted." 23.*

---

* Ed. Journal, No. 1, p. 23.

The operation was performed at mid-day. Nothing particular occurred till the evening, when so much pain was complained of, that Mr. S. loosed some of the stitches, and allowed some clots of blood to escape. He was very low in the night, with occasional vomiting, which continued next day, till an opiate injection was prescribed. On the 3d day the wound was examined, and looked well. The opiates were dispensed with after the first week—the bowels were brought into a good state by the occasional use of turpentine injections—the appetite—sleep—pulse, continued to improve—in short, every thing went on well, till about a month after the operation, when symptoms of ascites made their appearance, and, in spite of all their efforts, carried the unfortunate patient to his grave, after his heroic sufferings. On dissection, the liver was found altered in structure, and much enlarged. The spleen was greatly enlarged. We are ready to accord our humble mite of praise to the boldness and dexterity of the operator and his able assistants. They deserved success—in fact, we consider the *operation* as perfectly successful. The final event no human power could control.

---

3. *Amputation.* Mr. Syme, of Edinburgh, who so successfully amputated at the hip-joint, has offered some remarks on amputation generally, in the last number of our respected cotemporary of the North. If the following be the present practice of the Edinburgh surgeons, a censor in that metropolis appears necessary. "The manner of proceeding here, as every surgeon knows, is, first, to dissect back the skin *for some inches*—then to cut the muscles as high as they are exposed—and, lastly, to remove the bone as far up as the retraction of the flesh will allow." We can only say that no such practice is followed by any good modern surgeon on this side of the Tweed. Twenty or thirty years ago, indeed, we have seen the skin and cellular membrane turned up *about an inch and half*, like the cuff of a coat, and then the muscles divided down to the bone, by one circular incision. But this practice has long been abandoned. In the thigh, for instance, the circular incision reaches the muscles, and then the integuments retract, and are assisted in retraction—but not separated by dissection from the muscles underneath. Another circular incision, with the edge of the knife slanting upwards, divides the superficial muscles. These being allowed to retract, a third slanting circular is made to the bone, which is insulated an inch or two, and then sawn through. The limb thus removed presents the form of a cone, with a cylinder of bare bone at the apex. It is manifest that, in this way, an excellent cushion of muscle, and a sufficient covering of skin are left for the formation of a good stump. Such is the English practice, and certainly it forms a great contrast with that of Edinburgh, as represented (we hope somewhat in caricature) by Mr. Syme. The practice of the French surgeons—at least of their head—M. Dupuytren—is still worse than the Scotch. "M. Dupuytren cuts all the soft parts at once to the bone, which he next removes, after retracting the muscles to what he considers a sufficient extent. But here he is not particular, as on most occasions he makes no attempt to obtain union by the first intention. Indeed I have seen him dress the bone all round with dry *charpie*, previously to *approximating* the edges of the stump, for it was seldom possible to bring them into contact." After condemning all forms and modes of amputation by circular incisions, our author goes on to recommend the flap operation. This operation, we are aware, is very useful and even necessary on some occasions, as in the leg and at joints: But we doubt much whether it will ever come into universal practice in every kind of amputation, as Mr. S. recommends. We shall give Mr. Syme's directions, however, on the subject.

"Lisfranc recommends a long very narrow knife, sharp on both edges; but I think the one used by Mr. Liston better calculated for the purpose. It is about six inches in length, and five-eighths in breadth, thin and blunt in the back, except for an inch from the point which is very sharp; the back is straight and so is the belly, except about an inch and a half from the point, which is slightly convex.

"The dimensions which I have stated are fully sufficient for the arm and fore-arm, the leg, and all amputations in children; for the thigh of an adult, a greater length will of course be required.

"The surgeon grasping the limb with the left hand, at the place where it is to be removed, ascertains the situation of the bone by means of his thumb. He then introduces the knife over the bone at that part where he wishes to apply the saw, and, perpendicularly to it, he passes close by its side, and so on until the point appears directly opposite.* This finishes the first part of the operation, or the transfixion, as it is called; after which, he cuts his way outwards, in a line forming an angle with the bone more or less acute according to circumstances, so as to complete one flap.

"He then embraces the remaining undivided parts with his left hand, and, gathering them together, passes the knife on *their* side of the bone, so as to insulate it completely. Removing the restraint of his left hand, he now forms a second flap in the same way as he did the first.

"If there is only one bone, he immediately divides it, his assistant holding aside the flaps with his hands as high as it is exposed; if there are two, he separates the interosseous substance, and does the same thing; thus finishing his operation, which, with reasonable haste, and without the smallest hurry, need never occupy more than half a minute at most."—*Ed. Journal.* 38.

For many good remarks on removing fingers, toes, &c. we refer to Mr. Syme's paper.

---

* "There is some difference of opinion as to whether this first flap should be formed from the external or internal side of the limb. But as this seems to me a matter of perfect indifference, unless in reference to the operator's convenience, I would advise the surgeon, unless he be ambidexter, always to pass the knife, in the first instance, on that side of the bone which corresponds with his right hand as he stands before the patient."

4. *Rupture of the Axillary Artery.** The reduction of old luxations is not always unattended by danger as well as pain. A man, 50 years of age, had his left shoulder dislocated by the fall of a heavy chest on the part. A physician attended and pronounced the accident to be *a fracture of the humerus just above the elbow!!* Bandages and splints were accordingly applied, and in *two weeks* the fracture was declared so firmly united as to require no farther dressings. Soon after this the luxation at the shoulder was discovered by another physician, and an unsuccessful attempt made to reduce it. Two months after the accident, the patient came under the care of Professor Gibson, of Pensylvania. The reduction was effected on the 12th May, by venesection and pullies, after great force had been applied in various ways. A general swelling soon took place about the deltoid and pectoral muscles, but this was not, at first, considered to be of any consequence. But the tumefaction increased, and the patient died the same night. On dissection it was found that the axillary artery was torn directly across, near the glenoid cavity of the scapula, and great quantities of blood effused among the cellular membrane of the neighbouring parts.

The records of surgery, Professor G. observes, furnish very few examples of injury, much less death, from the reduction of old dislocations. Dessault relates the history of one case where either a large emphysematous or bloody tumour formed under the pectoral muscle immediately after the os humeri had been restored to the glenoid cavity. On the 13th day, this tumour had entirely disappeared. No case of the rupture of the axillary artery could be found on record, by Professor G. except the mention of such an occurrence by Charles Bell, who says, that this accident took place at the Newcastle Infirmary, obliging the surgeons to immediately amputate the limb. The extreme rarity of such an unfortunate event authorizes us to make attempts at reduction uninfluenced by the chance of its occurrence. But a remark has struck us respecting the cases of Professor Gibson, and Mr. Charles Bell, which we shall submit to our surgical brethren. In Mr. Gibson's case, one of the means of reduction was a ball placed in the axilla, while all the force which he could exert with his heel was applied by the surgeon. Having been unsuccessful, one of his assistants, Mr. Strudwick, tried the same plan, but failed. Again, in the case mentioned by Mr. Bell, the *Ambe* was used in the Newcastle Infirmary. Thus, in both these instances of rupture of the axillary artery, great pressure was necessarily made in the axilla. Was not therefore the artery cut across by *pressure*, rather than ruptured by *distension?*—we think the former infinitely the more probable of the two—and if so, it is a reason why we should be loath to have recourse either to the ambe or the ball in the axilla, for the reduction of dislocations of the shoulder.

---

* Philadelphia Journal, No. 13.

5. *Extravasation within the Cranium.** A man, 55 years of age, fell from a scaffold, and pitched with his head on the pavement. He was taken up, not insensible, placed in a fiacre, and carried home. There he soon complained of weight in his head, and pain in his hands. About 8 ounces of blood were taken from the arm—he presently lost the power of speech, and he was conducted to the Maison de Sante. *Symptoms*, five hours after the accident; abolition of the intellectual faculties and sensorial functions—inability to raise the eyelids—dilatation of the pupils—breathing free—pulse natural—involuntary discharge of urine—deglutition unimpaired—incomplete paralysis of the members of the left side—two wounds of the integuments of the cranium, one above the *right* ear, the other on the *left* side of the occiput. He was a second time bled, and evinced some return of sensibility, but soon relapsed again.

Next morning, M.M. Beclard et Dubois examined the patient. M. Beclard pronounced that there was extravasation within the cranium, on the right side, under the small wound, the paralysis being on the opposite side. The crown of the trephine being applied, and the osseous disk removed, a coagulum of blood was discovered an inch in depth, the limits of which could not then be ascertained. Two more discs were removed by the trephine, and the apertures brought into one. The extent of the clot was then ascertained, and found to be very considerable, being four or five inches in diameter. At different times during the operation, the patient gave signs of great impatience. On the internal surface of the third disc of bone removed, there was a fracture, and a projection or spicula of bone. No indication of this fracture was apparent on the outside of the skull. There was a wound of the meningeal artery, whence vermillion blood was still issuing. As the man was far from being enfeebled, the hæmorrhage was suffered to proceed, no compression of the brain being now feared. By different means the extravasated blood was removed. After the operation, and when placed in his bed, the patient entirely recovered his sensibility, and began to move the hitherto paralyzed members of the left side. He then fell into a light slumber, from which he frequently awoke, demanding something to eat. He was without fever, and complained rather of pain in the shoulders and hands than in the head. Next day, the dressings were removed, and the dura mater had risen nearly to a level with the interior aperture. The coagulated blood was almost entirely disgorged. The wound was dressed daily. By the fourth day, suppuration was established— no stool since the accident—gentle laxative, and a glyster—a scanty stool—lancinating pain in the left breast—fulness and frequency of pulse. Sixth day, the pain dispersed—the patient greatly incommoded by the pulsations about the wound. These on the 7th day were greatly increased, with pyrexia and loss of appetite, constipation of the bowels. *Lavements.* 8th day, hardness of pulse, somnolency, yellow tint of skin and eyes, some delirium in the evening, and tendency to coma. Venesection. 9th and 10th days,

* M. M. Beclard et Dubois, fils. Archives Generales, Nov. 1823.

sensibly better. The dura mater covered by fleshy granulations, follows the cerebral pulsations. From this time the recovery was rapid and uniform.

We consider that the patient ran a very great risk from the neglect of the bowels, and of sanguineous evacuations. Antimonials were prescribed, which served in this case as a substitute for more active depletion.

---

6. *Fracture of the Cranium.** Prompt surgical assistance often rescues the patient from the very jaws of death. Mr. Baker, of Staines, relates the case of a boy whose cranium was twice fractured with depression, and twice trephined in the course of two years. By the kick of a horse, on the 14th of August last, the left parietal bone was fractured, depressed, and the dura mater torn—there was also a wound on the scalp, with insensibility, and paralysis of the right arm. The trephine was employed, and the depressed portions of bone removed. There were many bad symptoms, but by purgatives, and afterwards antimony and small doses of opium, the fever was reduced. Two fungi cerebri arose, one as large as an egg. Compresses dipped in a mixture of rectified spirit and water, and kept on the fungi caused them to slough, and the patient perfectly recovered. This was the second accident of a similar kind, and requiring a similar operation in the same subject. The case is creditable to Mr. Baker.

---

7. *Tourniquets in Amputation.* The force and velocity of the arterial circulation are greatly over-rated even to this hour; but the danger of hæmorrhage in amputation is gradually losing its influence over the minds of surgeons, notwithstanding the glowing colours in which it has been painted by the late John Bell. In amputating at the shoulder joint and high up in the thigh, we fearlessly operate without a tourniquet—why not do the same when the removal of the limb is lower down, and when, of course, the vessels cut are smaller?—When a tourniquet is applied, we do not arrest the circulation in both arteries and veins at the same time. It is evident, from the engorgement of the limb, that the parts below the tourniquet contain *a great deal more blood* than they did prior to its application. All this is lost to the patient—and it is far more, we think, than would be lost in amputating without the tourniquet; for by compressing the artery with the finger we diminish the arterial circulation, without offering any impediment to the return of blood by the veins. Hence, in this way, the limb to be removed contains *less blood* than natural. After the removal of the limb there is generally more blood lost while screwing and unscrewing the tourniquet, than while tying the vessels without the aid of more pressure than the fingers can supply. These arguments and objections are well maintained in a short paper on the subject, by that able and bold operator Mr. Liston, of Edinburgh, in the last number of our respected cotemporary—the *Ed. Journal.*

---

* Mr. Baker, Med. Repos. No. 2, New Series.

8. *Ligature of the Subclavian Artery.** This formidable operation has so seldom been performed successfully, that we are bound to notice every instance terminating well. A sailor, 60 years of age, was admitted into the Lynn Dispensary, on the 31st of March, 1823 —with a soft pulsating tumour, " extending obliquely from the sternal end of the third rib, to a little above, and within one-fourth of the humeral end of the clavicle." In a consultation it was determined to operate. We need not detail the steps of the operation. Nothing particular occurred till the 16th day, when hæmorrhage took place, and recurred several times afterwards. The most remarkable circumstance, however, was, that after this the aneurismal tumour began to increase, and was evidently suppurating, when, in a paroxysm of coughing, six or eight ounces of bloody pus were brought up. The tumour was suddenly diminished one-half. It was punctured, and five ounces of the same kind of matter were discharged. " A cavity could now be felt between the first and second ribs, at their sternal ends, through which the fluid had passed into the lungs; and as there was now a free communication with the lungs, the air passed freely into the sac whenever he coughed, distending it, and sometimes escaping by the external wound."

From this desperate state the patient recovered—then had hæmorrhages—attacks of erysipelas, &c. but ultimately escaped from all. The case is very creditable to provincial surgery.

9. *Varicose Veins.* On the 13th of November, 1823, M. Richerand read a short paper, at the Academy of Surgery, on the cure of varicose veins. For some time past the professor has been in the habit of curing this state of the vessels by a simple longitudinal incision of some inches in length, applying charpie to the wound to prevent healing by the first intention, and to secure suppuration. By this operation the vessels are emptied of the partially coagulated blood with which they are filled, and become entirely obliterated, without any inflammation spreading along the internal surface of the vessels, as is sometimes the case when they are tied or simply cut across. The pain of the operation is by no means severe. M. Richerand refers the Academy to cases which have been cured in this manner by himself and others.

10. *Disagnosis of Fractures.* M. Lisfranc has extended the application of the stethoscope to fractures, assuring us that, by this instrument no surgeon can remain in doubt respecting the existence of a broken bone in any part of the body except the head, however considerable may be the degree of tumefaction around the fracture. The instrument is to be applied over or near to the seat of the accident, when the least motion of the part will produce a distinct sound through the stethoscope, especially with the end piece out. When the bone rides the sound is less obvious. The crepitation of compact bones

* Mr. Bullen. Med. Repos. No. 117.

gives a sharp, strong, crackling sound—that of spongy bones resembles the action of a file on such a substance as pumice stone. Oblique fractures crepitate more distinctly than those that are transverse. If liquids are effused round the fractured extremities a sound is superadded like that of the foot thrust into an old shoe soaked in water. When the fracture is complicated with splinters the crepitation is united with a crackling, as of several cornered bodies rubbing on each other.—*Archiv. Gen. Aout.* 1823.

11. *Tracheotomy.** Mr. Liston has lately performed the operation of tracheotomy in two cases—one for œdema glottidis, and the other for injury of the larynx. The case of œdema glottidis was one of pressing danger, and where suffocation was impending. Considerable difficulty was experienced in keeping the tube in the trachea, and the necessity for its presence there has continued ever since, as the aperture of the rima glottidis is not of such dimensions as to enable the patient to dispense with the tube. This case bears the nearest resemblance to that of Mr. Price (related in a former number of this Journal) of any on record. Mr. Price has now breathed through the tube nearly eight years, and is in good health at Portsmouth.

12. *Femoral Aneurism (supposed) cured by spontaneous Rupture of its Sac.*† Petro Valentine, 61, strong and vigorous constitution naturally, but worn down with age, indigence, and debauchery, was attacked with jaundice, at a time when he was experiencing much inconvenience from an increasing weakness and pain in the left leg. This was in May 1820. The jaundice yielded; but the constitution remained low, and the diseased leg had assumed a sallow and doughy appearance from the thigh downwards, being œdematous about the ancle and foot. Upon examination, a flat circumscribed tumour, better than an inch in diameter, was visible about the middle of the thigh, on the inner side of the sartorius muscle, and immediately over the femoral artery. The pulsation was visible and tangible—its contents fluid, and yielding to pressure, but immediately resuming their place again. This tumour was first perceived about two months previously by the patient. In consultation it was unanimously pronounced to be a case of true femoral aneurism. In consequence of the state of the patient's health the operation was delayed; but the limb became more swollen, and the health rapidly declined. The operation was therefore determined on; but on the night before it was to be performed, "*the patient experienced an instantaneous and violent pain in the knee, attended with considerable noise, proceeding from the rupture of the tumour. An immediate distention of the whole limb ensued, accompanied with an extensive ecchymosis from the glutei muscles inclusively, down to the knee, giving it a dark livid colour. The tumour entirely disappeared.*" The patient was much debili-

* Mr. Liston. Edinb. Journal, 77.

† Dr. Archer. American Medical Recorder, No. 24.

tated by the extravasation, and complained of great tension of the thigh—his pulse was feeble, and his spirits depressed. A compress and bandage were applied, and the limb bathed with spirits of camphor. This treatment was continued several days—but on the third, the limb was evidently smaller—the patient's health improving—and, finally, by the end of the third week after the rupture, the swelling and ecchymosis had so far subsided, as to permit him to walk about the room—and, in a month more, he was enabled to follow his usual avocations.

We are disposed to be sceptical as to the fact of the above being a case of true aneurism and bursting of the sac, followed by so easy and sudden a cure. The physician in attendance has said nothing of the phenomena of the circulation in the limb, either before or after this supposed rupture. The pulsatory sensation was no proof of aneurism—as a tumour of the vein situated in the course of the artery would exhibit the same phenomena. Indeed we are far more inclined to consider the case as one of venous than arterial disease and rupture.

---

13. *Removal of Elongated Uvula.** It is now known that the constant irritation of an elongated uvula will induce symptoms resembling chronic inflammation of the chest. The following is a case of this kind.

Madam G. 30 years of age, healthy from her birth till January 1822, when she became affected with slight cough, to which little attention was paid. It increased, however, till April, when the ordinary medical attendant was consulted, who prescribed emulsions, &c. without benefit. In the month of August the symptoms had made such progress that the patient was pronounced to have chronic pneumonia accompanied by tubercles, and was treated accordingly. In the month of December M. Cursnat was consulted, and observed the following symptoms, viz.—tightness of the chest in breathing, accompanied by sharp but fugitive pains in the thorax during coughing or making a deep inspiration—constant efforts to clear the throat of mucus—fixed pain in the larynx—loss of appetite—countenance pale—great emaciation. Yet the thorax sounded well in every part, except near the top of the sternum, whence a dull sound was emitted. The pulse was small and unequal. On examining the throat, the point of the uvula was seen lying on the root of the tongue in a relaxed and elongated state. A portion of the uvula was therefore removed by a pair of scissors, and the discharge of blood and mucus soon restrained by an astringent gargle. In a fortnight the whole of the abovementioned symptoms had entirely disappeared, and she soon acquired health and strength.

---

14. *Acupuncture.*† Mr. Churchill, who published some time ago on this process, informs us that—" its success has now been so con-

---

* Revue Medicale, tom. xi. † Mr. Churchill. Med. Repos. 113.

spicuous, that he can assume an air of triumph, and dare any one to express his disbelief in what he has asserted respecting it." However successful and triumphant may be acupuncture, the above is not the language to announce it in. But it is the language of youth, and time will chasten it. Mr. Churchill has here brought forward three cases of rheumatism cured, or at least greatly relieved by this curious process. The first was a gardener, who had been rheumatic for three or four years, in consequence of exposure to wet and cold. The neck, shoulders, back, and hips, had been occasionally the seat of the pain. In the beginning of the present year it lost its erratic character, and became fixed in the deltoid and pectoralis major of the left side. There it resisted all kinds of remedies. "A needle was introduced about midway between the point of the shoulder and the insertion of the deltoid muscle, which pierced through the belly of this muscle until its whole length (one inch) had passed." The patient became sensible of relief before the needle had reached more than two-thirds of its depth, and when the whole way, the pain had entirely left the part. The needle was allowed to remain five minutes, when it was removed, and introduced below the clavicle, so as to pierce the pectoralis. This puncture was as successful as the former. It appears that the man went to work, with only debility of the parts, "and in a week or two felt no remains of the disease."

The second case was one of lumbago. Mr. C. introduced two needles, two inches in depth, into the muscles of the loins, "which in some degree lessened the violence of the pain in a minute or two." Finding that the disease was not removed but mitigated, he passed a third needle and a fourth into the lumbar mass of muscles, and in a few minutes the pain vanished. Mr. C. heard nothing of him for two days, when his daughter called to say he was quite well. The third case is rather equivocal—and at all events the pain had only commenced the same morning in a sudden manner. It might therefore as suddenly disappear under the mental and corporeal impression. We understand that the Earl of Egremont has derived considerable advantage from acupuncture; and therefore we should not be surprised if it become fashionable in rheumatism, which so often resists every means of cure. At the risk of coming under Mr. Churchill's censure we venture to doubt, not what he has said or done himself, but a similarity of success in the hands of others. Sincerely do we wish, however, that we may be wrong in our anticipations.

---

15. *Removal of an immense Tumour.** Mr. Liston we consider as one of the boldest operators of the present day. He lately removed a tumour weighing 44 pounds, involving the whole of the external genital organs, and descending below the knees. The hæmorrhage was tremendous, and compared by the bye-standers to the discharge of water from a shower-bath! Syncope took place, and the vessels were secured. The man recovered.

---

* Mr. Liston. Edinb. Journal.

16. *Acupuncture.** Dr. Tweedale has related a case exemplifying the utility of acupuncture in anasarca, where the cellular membrane of the upper and lower extremities and trunk was enormously distended with fluid, accompanied by cough and distressing dyspnœa. Various remedies had been tried without benefit. Acupuncture, with a common needle of moderate size, was easily performed, a piece of thread being several times passed round the instrument a quarter of an inch from the point. A dozen of punctures were made in each leg, with little or no pain. The result was most satisfactory. The arms and trunk were reduced, in the course of a week, to their natural size. The punctures were repeated several times. Dr. T. adds his testimony to that of Dr. Sutton and Mr. Finch, in favour of acupuncture.

Mr. Finch, of Greenwich, in the succeeding number of the Medical Repository, states a case of trismus approaching to tetanus traumaticus successfully treated by acupuncture. Mr. F. avers that he has had frequent opportunities of witnessing the benefit of this measure in chronic rheumatism, especially where there was rigidity of the muscles, and this led him to try it in the case before us. The patient was wounded and lacerated by falling from a considerable height, and trismus had supervened. The pulse was 130, the jaw completely locked, and deglutition rendered impossible. Mr. T. introduced a needle into the masseter muscle of the right side, and soon found that that and several other muscles about the neck and throat instantaneously relaxed. Another needle was then pushed into the opposite masseter, and relief (though not to the same extent) was forthwith afforded. In short, so rapid was the change, that before Mr. Finch left the room the patient was enabled to take a large dose of tincture of opium and a cup of chocolate. He perfectly recovered. The process deserves trial in all cases of this kind; because it can do no harm in the event of its doing no good.

---

17. *Subclavian Aneurism.* A man presented himself at the Royal Infirmary of Edinburgh on the 7th of August last, and in a fortnight after admission an aneurism of the subclavian artery was discovered. The pulsation soon became obliterated in that arm, and on the 23d of August the subclavian artery was tied by Mr. Wishart, without any difficulty. The patient bore the operation well, and expressed no uneasiness when the ligature was drawn. The man did well, excepting an erysipelatous attack of the arm, which suppurated. On the 16th day the ligature came away, and next day pulsation was felt in the radial artery of the arm. By the 24th October he was walking about, and the case may now be considered as having terminated successfully. " The success of the operation, says Mr. Wishart, and the short time in which it was performed may be ascribed to the method employed, viz. after making the external incision, and dividing the platysma-myoides, the laying aside the knife and using the fingers in separating the cellular substance, so as to expose the

---

* Dr. Tweedale and Mr. Finch, Nos. 118, 119.

artery. No difficulty was met with in passing the ligature, or drawing it sufficiently tight with the fingers." *Ed. Journal, No.* 1, *New Series.*

---

18. *Suppression of Urine.** This is very generally a fatal disease. Mr. Bidwell, of Warbleton, has related a case of recovery in a man about 60 years of age, who had been three days without making any water. He complained of great pain in the region of the *bladder*, yet no water followed the introduction of the cathether. He was plethoric, and had a hard jarring pulse at 90. He had been bled in the erect posture to nearly three pounds without producing syncope. He was immersed in the warm bath—had a grain of digitalis every three hours, with a draught of camphor mixture, and sp. æth. nit. Fourth day, was much relieved, but still made no water. He was cupped over the lumbar region to the extent of *three pounds*, and was ordered one grain of calomel, and two of digitalis *every three hours*, together with ℥iss. of the infusion of digitalis at the same periods. These were tremendous doses, if the digitalis was good for any thing. After two days of this treatment, he was reported to have singultus, tendency to coma, and sinking pulse. The urinary secretion was still suspended. The digitalis was now omitted, and a camphorated æther mixture substituted. Large quantities of water were now discharged, and the patient recovered. We know not what to say to this case. Our readers must judge for themselves.

---

19. *Retention of Urine.*† Mr. Holbrook invites the attention of his brethren to the effects of active purging in cases of obstruction to the flow of urine from the bladder. As the more usual causes of retention of urine are affections of the prostate gland in old people, and strictures of the urethra in all ages; and as the exciting causes are generally cold applied to the body, and excesses in drink, Mr. Holbrook concludes, that a spasmodic state of the muscles surrounding the urethra exists, with probably a relaxation of the bladder itself, and a fulness of the vessels about the neck of the urinary organ.

"In these cases, I have commonly found the patients complaining of pain and tension about the lower part of the belly; pain across the loins, with desire to pass urine, without the power; sickness; some degree of fever; frequently stupor, particularly in elderly persons. A common practice in these cases I know to be, if the catheter fail to be introduced, the immediate recourse to the warm bath, the exhibition of an opiate, and perhaps an opiate glyster; and if this fail, some leeches are then applied to the perineum; or, if the patient be of a full habit, blood is perhaps taken from the arm. All this time, the bowels are often totally neglected, or if any thing is done in that way, a little castor oil only has been exhibited, under the mistaken fear of adding to the irritation by more active medicines, and thus, in

---

* Mr. Bidwell. Med. and Phys. Journ. 284.
† Mr. Holbrook. Med. Repos. No. 112.

these cases, as well as in many other surgical diseases, the attention is too much confined to the local affection. Patients under this treatment certainly do frequently recover, but not, in general, until the whole system has been greatly exhausted, and relaxation of the spasm is in consequence produced. Frequently, in these cases, the urine follows the withdrawing of the catheter, after an attempt at its introduction, which, by producing the sensation of the passage of the urine, removes the spasm, owing to the sympathy which exists between the fore part of the urethra and the neck of the bladder. If, then, the urine has been known to pass from the sympathetic effect of simply withdrawing a bougie or catheter from the fore part of the urethra, how much more powerful must be the effect of calling into action all the combined powers for evacuating the contents of the bowels, which are so much accustomed to act together with the urinary organs, that we find it impossible in the healthy state to evacuate per anum, without also, at the same time, emptying the bladder? Instead, therefore, of trusting solely to the above soothing mode of treatment, if recourse is immediately had to a full dose of calomel, combined with a little extr. papav., followed by a purgative mixture, repeated every two or three hours, until the bowels are thoroughly cleansed, both from the residue of the ingesta and foul secretions (some blood being previously taken from the arm, if the patient be of a full habit, or symptoms of inflammation appear), I am convinced, from repeated experience, that complete relief would, in most instances, be procured in a few hours, provided too much time has not been lost before the means was employed, and, even then, a better chance will be afforded for the full effect of other remedies. Under these circumstances, the warm bath should be had recourse to, and, whether bleeding has or not been premised, leeches should be applied to the perineum, frequent injections of glysters of warm water used, and opium administered, both internally and in the form of glyster, mixed with milk. When, from long distention, the bladder has entirely lost its power of contraction, if the catheter cannot be introduced, there only remains to puncture the bladder."

---

20. *Taliacotian Operation,** Mr. Davies, of Tottenham Court-road, has been successful in restoring "the human face divine" to a shoemaker, who had lost his nose and great part of the upper lip, for more than three years by syphilis. The operation was performed at the Saint Pancras Work-house, on the 19th September, 1823, in the presence of Dr. Roots, Mr. Barrack, and other medical gentlemen. The operation was very complicated and difficult—and, from all accounts, remarkably successful. Mr. Davies is entitled to great praise in accomplishing the task which he imposed on himself.

---

* Mr. John Davies. Repository, No. 1.

